# Abundance and Distribution of Korean Flower Flies (Diptera: Syrphidae): Dominant Influence of Latitude on Regional Distribution

**DOI:** 10.3390/insects11040213

**Published:** 2020-03-29

**Authors:** Tae-Sung Kwon, Cheol Min Lee, Ok Yeong Ji, Mohammad Athar, Sung Cheol Jung, Jong-Hwan Lim, Young-Seuk Park

**Affiliations:** 1 Alpha Insect Diversity Lab, Nowon, Seoul 01746, Korea; insectcom@naver.com; 2 California Department of Food and Agriculture, 2800 Gateway Oaks Drive, Sacramento, CA 95833, USA; leecheolmin77@gmail.com (C.M.L.); athar.tariq@cdfa.ca.gov (M.A.); 3 Korea Fly Laboratory, Gangdong, Seoul 05268, Korea; okjie@hanmail.net; 4 Division of Forest Ecology and Climate Change, National Institute of Forest Science, Dongdaemun, Seoul 02455, Korea; scjungkr@korea.kr (S.C.J.); limjh@korea.kr (J.-H.L.); 5 Department of Biology, Kyung Hee University, Dongdaemun, Seoul 02447, Korea

**Keywords:** local distribution, regional distribution, global distribution, occupancy, abundance, species diversity, one-dimensional distribution, hierarchical scale

## Abstract

Studies on abundance and distribution at different scales are rare. We examined whether the abundance of flower flies at a site in South Korea was related to the national occupancy and global distribution (distributional extent or range size) and whether the national occupancy was related to global distribution. In global distribution, the influence of two dimensions (latitude and longitude) was analyzed separately. Flower flies were collected by malaise and pitfall traps at a forest gap in South Korea. Data regarding national occupancy and global distribution were obtained from a Korean Flower Fly Atlas. We collected 46 species from the field survey and obtained a list of 119 species from the Korean Flower Fly Atlas. Our results showed that abundance at a site was positively correlated with national occupancy, but not global distribution, and the national occupancy was positively correlated with global distribution, mainly by the latitudinal range size. Finally, our results indicated that the regional distribution of flower flies was influenced by its one-dimensional global distribution.

## 1. Introduction

Abundance and occupancy relationships comprise some of the most general and well-explored patterns in ecology [1,2,3]. Positive correlations between abundance (i.e., number of individuals collected) and occupancy (i.e., number of sites where collections are made) were reported globally, including in terrestrial, marine, and freshwater biomes for various taxa, such as birds, butterflies, flower flies, mammals, protists, and plants [1]. Therefore, this positive correlation between abundance and occupancy is one of a few general ecological patterns. Several hypotheses, such as measurement effects, structural mechanisms, dynamic mechanisms, spatial aggregation, and nonindependence, have been proposed to explain these correlation patterns [1]. Among them, Brown [4] proposed that a species with a wide niche also has large range and population sizes, whereas a species with a narrow niche has a narrow range size and a small population size. Metapopulation dynamics were also proposed to explain this correlation [1]. A species of high abundance has a higher chance of dispersal and occupies more sites compared with a species of lower abundance. However, the correlation can be simply explained by the null hypothesis under random distribution [5]. A low rate of detection for species with low density partly results in the positive correlation [4]. The positive correlation is also predicted by the neutral community model, which assumes that local communities are mainly built by the dispersal of species with identical ecological characteristics [6].

Therefore, a species with a wide distributional extent has a higher density compared with a species with a narrow distributional extent. In many cases, local assemblages are numerically dominated by widespread species [7]. Thus, it is very likely that the abundance of species in a local community is positively related to its distributional extent on a wider scale. However, this prediction is rarely investigated because most studies on abundance and occupancy examine them at the same distributional scale [8,9,10]. The abundance of birds in a region was positively correlated with the range size at the sub-continental scale [7]. Kim and Kwon [11] reported that the abundance of butterfly species at four sites in the same region in South Korea was strongly correlated with national occupancy. The abundance of flower flies in a site in the United Kingdom positively correlated with range size at the continental scale [12].

Flower flies are a diverse dipteran group whose larvae are phytophagous, saprophagous, or predators of aphids and other insects. The adults rank second only to bees in their importance as pollinators [13]. Some Diptera are notoriously difficult to identify, which hinders the ecological research of this group. However, unlike other dipteran families, flower flies have a distinct morphology and, thus, are relatively easy to identify at the species level. Therefore, flower flies are widely used for various ecological studies and for testing ecological theories and hypotheses [12,14,15].

Atlas surveys yield one type of regional occupancy data and allow us to examine the abundance and occupancy relationships in large-scale population dynamics [3,16]. Atlas data have become an indispensable tool for documenting distribution and populations for conservation purposes, providing a framework for survey design, assessing species–environmental associations, and generating hypotheses regarding the causes of range change [17,18,19]. Kwon et al. [17] reported that the northern margins of southern species shifted northward in response to climate change, whereas the southern margins of northern species shifted southward owing to habitat enlargement based on occurrence data from two Korean butterfly atlases.

In the present study, we determined whether the abundance of flower flies at a site in South Korea is positively correlated with national occupancy and the global distributional extent and whether national occupancy is positively correlated with global distributional extent based on a Korean Flower Fly Atlas [20]. Global distributional extent includes the two dimensions of latitudinal and longitudinal range sizes. These two dimensions have different ecological aspects. In the northern hemisphere, latitudinal range size is closely related to the temperature range of each species, whereas longitudinal range size is related to natural dispersal, biogeographical history (e.g., location of origin), continental drift, and anthropogenic dispersal, such as biological invasions through local or international trade [21,22,23,24]. Therefore, the two types of range size influence regional or local fauna differently. To our knowledge, this expectation has not previously been examined. For the first time, we tested this hypothesis based on the data of the present study.

## 2. Materials and Methods

### 2.1. Data Sampling

We obtained abundance data for flower flies (Diptera: Syrphidae) from a site in South Korea through a field survey; data on their distribution at the global scale, as well as occupancy at the Korean nationwide scale, were retrieved from a Korean Flower Fly Atlas [20]. We collected flower flies at a large forest gap (~27,742 m^2^) in the Gwangneung forest (N37°45′60″, E127°10′46″) in the mid-western part of the Korean Peninsula using standard malaise traps (width 180 cm, length 165 cm, height 110–176 cm) and pitfall traps (diameter 9.5 cm, depth 6.5 cm). Malaise traps are widely used for collecting flower flies [12,25]. Although pitfall traps are rarely used for collecting flies, Lee et al. [26] reported collecting a diverse range of flies with pitfall traps. Annual precipitation in the study area is 1364.8 mm, and the annual average minimum and maximum temperatures are 3.7 °C and 16 °C, respectively (http://www.kna.go.kr).

The forest gap was formed in a forest of Korean pine trees (*Pinus koraiensis* Sieb. et Zucc.) in South Korea by the strong typhoon, Kompasu, in 2010 [27] and was replanted with young trees of Korean pine in April 2013 [28]. The gap is surrounded by Korean pine trees and deciduous trees (mainly *Quercus serrata* Murray). Three sampling sites (gap, edge, and forest) were selected along each of three lines in the forest gap (see Kwon [29] for details). The edge sites were on the boundary between the gap and the forest, whereas the gap sites and the forest sites were about 20 m away from the edge sites. Therefore, samples were collected from a total of nine sites (three forest, three edge, and three gap sites).

At each sampling site, one malaise trap and five pitfall traps were installed. The pitfall traps consisted of plastic cups (diameter 9.5 cm, depth 6.5 cm), linearly installed at 2-m intervals at each sampling site. Each trap was filled to ~20% with ethylene glycol as a conservation agent. Sampling by malaise traps was conducted once every two weeks from July 15, 2014 to December 4, 2014 and from June 19, 2015 to November 20, 2015. Samplings by pitfall traps were conducted biweekly from September 15, 2013 to November 30, 2013 and from July 15, 2014 to December 4, 2014. Collected flower flies were identified based on Han and Choi [20].

### 2.2. Data Analysis

We obtained data on the national occupancy in South Korea (hereafter referred to as occupancy) and the global range size from the Korean Flower Fly Atlas [20]. The number of recorded sites was considered to be the occupancy of each species. Longitudinal and latitudinal range sizes were quasi-quantitatively determined from the recorded nations or regions. Longitudinal range size was defined at four levels. Flower fly species recorded in a country of eastern Asia, such as Japan or China, were determined as level 1 in longitudinal range size, whereas those recorded in two or more countries in Asia were determined as level 2. Species recorded from Asia to Europe were determined as level 3 in longitudinal range. The Australasian, Oriental, and Afrotropical regions were considered to be within the Asia–Europe range size. The species recorded in the Asia–Europe range and in the Americas were determined as level 4 in longitudinal range size.

Latitudinal range size was defined at three levels. Species recorded only in temperate regions, such as Korea and nearby countries, such as Japan and China, were determined as level 1. Species recorded in warm (tropical or sub-tropical regions, such as South Asia) to temperate regions or in temperate to northern-temperate regions, such as Siberia, were determined as level 2. Species recorded in warm to northern-temperate regions were determined as level 3. The global range size of each species was estimated by multiplying the longitudinal and latitudinal sizes (1–12 levels). Abundance was defined as the number of individuals of each species collected during the sampling period and local occupancy was defined as the number of sampling sites in our field survey where each species was collected.

Differences in occupancy (i.e., number of recorded sites) were compared among categories of latitudinal, longitudinal, and global range sizes through an analysis of variance with the package stats in R [30]. When the occupancy was significantly different among categories, Tukey’s multiple comparison test was conducted with the laercio package [31] in R. Multiple regression analysis was used to find the relationship between dependent variables (abundance, local occupancy, and occupancy) and independent variables (occupancy and range size). Abundance and occupancy were log-transformed with natural logarithms before regression to improve the normality of variance. The regression analysis was conducted with the function lm in the stats package in R. We used Akaike’s Information Criterion (AIC) to evaluate the strength of the regression models.

## 3. Results

We compiled a list of 119 species recorded from the field survey and specimens housed in museums (Table S1), including two rare species (*Pipiza inornata* and *Xylota ignava*), which were first collected by our field sampling in South Korea. From the field survey in the forest gap, 46 species were collected (Table S2). Most flower flies were collected in open habitats, such as gaps (35 species, 919 individuals) and edges (29 species, 263 individuals), but a few flower flies were collected in forests (3 species, 7 individuals). Malaise traps and pitfall traps collected 42 and 12 species, respectively. However, four rare species (*Betasyrphus serarius, Eupeodes luniger, Ferdinandea cuprea*, and *Rhingia laevigata*) were collected only by pitfall traps.

**Figure 1:**
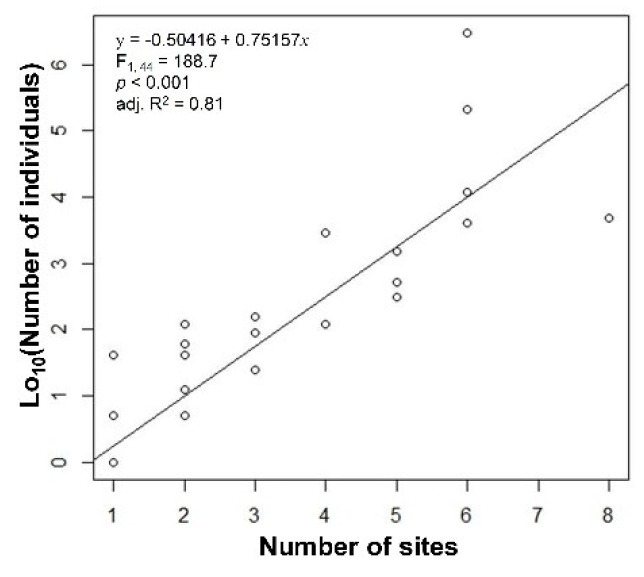
Abundance and local occupancy of flower flies at sampling sites in the Gwangneung forest gap, South Korea.

**Figure 2:**
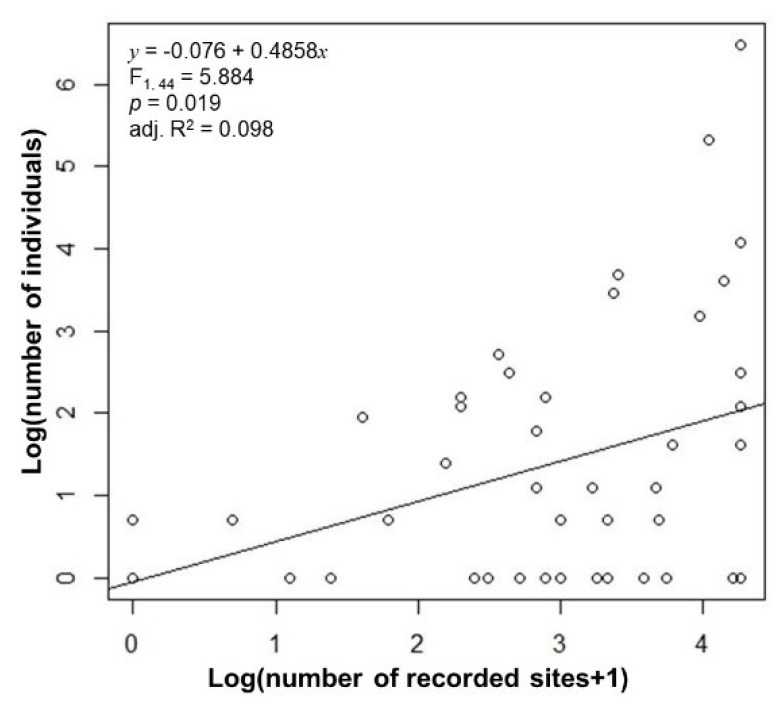
Abundance and national occupancy of Korean flower flies. The occupancy is considered to be the number of recorded sites in the Korean Flower Fly Atlas (Han and Choi 2001).

Abundance and local occupancy were significantly correlated (F_1,44_ = 188.7, adj. R^2^ = 0.807; Figure 1), so that their response to occupancy and global range size was similar (Table 1). Multiple regression showed that abundance was significantly explained only by occupancy but not by the longitudinal and latitudinal range sizes, nor by the global range sizes (Table 1). The same was observed for the local occupancy. A simple regression model using occupancy as an independent variable explained ~10% of the total variance. This simple model had a higher R^2^ value (0.098) than two multiple models (0.085 and 0.077), showing that the global distributional extent did not influence the abundance of the extant species in a local community. The simplest models had the lowest AIC values. These models had one independent variable for abundance, local occupancy, and national occupancy. The model that used latitude for national occupancy had a higher R^2^ and lower AIC compared with the model that used global range size, indicating that this was the optimal model. The relationship between abundance and occupancy is visualized in a scatter plot (Figure 2).

**Figure 3:**
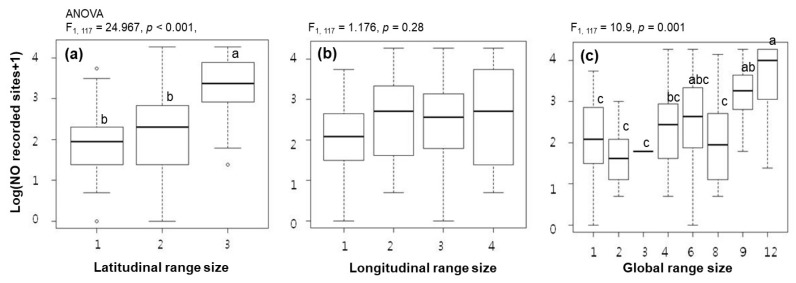
Boxplots of occupancy (national) and latitudinal range size, (**a**), occupancy and longitudinal range size, (**b**), and occupancy and global range size, (**c**), of Korean flower flies. Occupancy was considered to be the number of recorded sites in the Korean Flower Fly Atlas (Han and Choi 2001). Categories of 5, 7, 10, and 11 in global range size are not included in the figure because there were no recorded data. Scales of latitudinal, longitudinal, and global range sizes are defined in the text. The different letters on the boxplot indicate significant differences among range sizes based on Tukey’s multiple comparison test (*p* < 0.05).

The regression analysis between occupancy (regional distribution) and range sizes (global distribution) showed that only the latitudinal range size significantly influenced occupancy in the model containing the two dimensions of global range sizes (R^2^ = 0.177, *p* < 0.001) (Table 1). The longitudinal range size returned a negative regression coefficient with no significance. The global range size (multiplying two dimensions) was significantly related to occupancy, but its influence (R^2^ = 0.077) was lower than that of the latitudinal range size (R^2^ = 0.169). The occupancy increased as the level of latitudinal range size increased (F _1, 117_ = 24.967, *p* < 0.001) (Figure 3a), whereas it was relatively similar among the levels in the longitudinal range size (F_1,117_ = 1.176, *p* = 0.28). The occupancy increased as the global range size increased (F_1, 117_ = 10.9, *p* = 0.001) and Tukey’s multiple comparison test varied from small (1, 2, and 3) to large (9 and 12) sizes.

## 4. Discussion

### 4.1. Local and Regional Occurrence

Although 173 species of flower flies are recorded in the Korean Flower Fly Atlas [20], only 117 species were identified using museum and field specimens [20]. We collected two species (*P. inornata* and *Xylota ignava*) from our field sampling that were not recognized by authors of the Korean Flower Fly Atlas. Therefore, the final list used in this study contains 119 species (Table S1). Through field sampling, we collected 46 species in the Gwangneung forest gap (Table S2). The species richness (46 species) was higher than that reported by other studies on local fauna in South Korea, which ranged from 1 to 40 with 16.2 ± 12.3 (mean ± SD) [32,33,34,35,36,37]. However, this species richness was lower than the 67 species collected at the Wonju Yonsei University campus, which was extensively sampled by the Atlas’ authors [20]. Thus, our sampling is not sufficient to compile the complete list for a site.

Flower flies were previously sampled in open habitats, such as meadows, gardens, forest roads, and forest edges, rather than in closed habitats, such as forests [12,14,25]. In our survey, most flower flies (99.4% abundance, 100% species richness) were collected in open habitats, such as clearings and forest edges (Table S2). Gittings et al. [25] reported that, in Ireland, nearly 80% of Syrphid species are associated with open space habitats, rather than closed-canopy forest. However, in a Mediterranean landscape in Spain, the species richness of flower flies was higher in woodland than in scrubland and grassland habitats [38]. Woodlands comprise not only mature trees that provide a micro-habitat for rare species, such as saproxylic hoverflies, but also temporary or permanent bodies of water; thus, small scrub and grassy clearing areas provide extra resources for hoverflies [38].

The positive correlation between the local abundance of a site and regional occupancy agrees with the results of Owen and Gilbert [12], in that the abundance of flower flies at a site in the United Kingdom was positively correlated with the continental range sizes in Europe. However, the strength of the relationship in our study (R^2^ = 0.064, *p* = 0.05) was much lower than that reported by Owen and Gilbert [12]. European occupancy data are more robust than Korean occupancy data because Korean data rely on museum specimens and personal collections. This could explain the difference in R^2^ between our study and that of Owen and Gilbert [12]. The positive correlation between abundance and occupancy at different scales was comparable with the findings of Kim and Kwon [11], in that the local abundance of butterflies at four sampling sites was positively correlated with occupancy. The authors found that a positive correlation occurs between local occupancy (occupancy in four sampling sites) and national occupancy and between local abundance and local occupancy. This result corroborates our findings.

### 4.2. Regional and Global Occurrence

The positive correlation between abundance and distribution has received attention from macro-ecologists for several decades [1]. The positive relationship between abundance and occupancy across different species is one of the most robust patterns [10]. However, some studies show either no correlation or a negative correlation between abundance and occupancy [39,40]. In many cases, distribution explains 20%–30% of the interspecific variation in abundance [41]. In the present study, however, only 10% of the variation in abundance was explained by the occupancy. This might be due to the different scales of abundance (local) and occupancy (region) and low quality of occupancy data. At the same scale (local), however, about 80% of the variation was explained by occupancy (Figure 1).

There are global gradients in the species richness of plants and animals, from high biodiversity in the tropics to low biodiversity in polar and high-mountain regions [9,42]. Latitudinal and longitudinal gradients determine the substantial geographic variation in biodiversity. In our study, of the two dimensions of global range size, only one dimension (e.g., latitudinal range size) significantly influenced national occupancy. To our knowledge, this one-dimensional influence has not previously been reported. Although the local abundance of flower flies at a site has no relation to global distributional extent, the regional abundance on a national scale would be positively correlated with the global distributional extent. If the national abundance of Korean flower flies is positively correlated with national occupancy (this is general, as noted above), it would mainly be influenced by latitudinal range size, as well as national occupancy.

In South Korea, the abundance of many species or families of ants, beetles, spiders, and flies exhibits standard bell-shaped curves along the temperature gradient in the whole region, indicating that temperature is a key factor in the distribution and abundance of common arthropods. Since poikilothermic animals, such as arthropods, frogs, and reptiles, are more influenced by the thermal environment than homoiothermic animals, such as birds and mammals, the one-dimensional influence of global range size on regional occupancy and abundance would be more common in poikilothermic animals.

## 5. Conclusions

We aimed to investigate: (1) the relationship between the abundance of flower flies at sites and the national occupancy and global distribution; and (2) the relationship between national occupancy and global distribution. We observed a positive correlation between occupancy (regional distribution) and latitudinal range size (global distribution), indicating that widespread flower flies in South Korea have wide temperature niches. This result indicates that the tolerance range in the thermal environment is a key factor in the distributional extent of flower flies in temperate regions.

## Figures and Tables

**Table 1:** Regression models of abundance, local occupancy, and national occupancy. Abundance, occupancy, and range sizes are detailed in Table S1.

Dependent Variable	Independent Variable	Coefficient	*P* (t-test)	adj. R^2^	AIC
Abundance	National occupancy	0.5566	<0.05	0.085	172.7
	Latitudinal range size (Lat)	−0.4439	ns		
	Longitudinal range size (Log)	0.2147	ns		
Abundance	National occupancy	0.491	<0.05	0.077	172.2
	Global range size (Lat × Log)	−0.006	ns		
Abundance	National occupancy	0.4858	<0.05	0.098	170.2
Local occupancy	National occupancy	0.68765	<0.05	0.093	188.9
	Latitudinal range size (Lat)	−0.85275	ns		
	Longitudinal range size (Log)	0.03561	ns		
Local occupancy	National occupancy	0.59422	<0.05	0.073	189
	Global range size (Lat × Log)	−0.11588	ns		
Local occupancy	National occupancy	0.4921	0.0502	0.064	188.5
National occupancy	Latitudinal range size	0.8774	<0.001	0.177	338.1
	Longitudinal range size	−0.1609	ns		
National occupancy	Global range size (Lat × Log)	0.1099	<0.01	0.077	350.8
National occupancy	Latitudinal range size	0.7502	<0.001	0.169	338.4

## Supplementary Materials

The following are available online at https://www.mdpi.com/2075-4450/11/4/213/s1, Table S1: Flower flies (Syrphidae, Diptera) in South Korea. Table S2: Flower flies (Syrphidae, Diptera) collected at the Gwangneung forest gap in South Korea.
