# Supplementary material for: Abundance and Distribution of Korean Flower Flies (Diptera: Syrphidae): Dominant Influence of Latitude on Regional Distribution
Source: Insects. 2020 Mar 29;11(4):213. doi: 10.3390/insects11040213 (PMC7240411; doi:10.3390/insects11040213)
Supplement: Supplementary file 1 [file insects-11-00213-s001.pdf]

Supplementary information

Table S1. Flower flies (Syrphidae, Diptera) in South Korea. Scales of latitudinal, longitudinal, global range sizes are explained in text. Occupancy (national) is the number of recorded sites in a Korean flower fly atlas (Han and Choi, 2001). Local (Loc.) occupancy and abundance are the number of sites and individuals collected at the Gwangneung forest gap (about 2.8 ha) in South Korea, respectively. The flower flies had been collected from 2013 to 2015 by Malaise traps and pitfall traps (see Table S2). Species are arranged following Han and Choi (2001).

Species	Abundance (Gwangneung)	Local occupancy (Gwangneung)	National occupancy (South Korea)	Global distribution range size		
				Latitude	Longitude	Global
Allograpta javana	3	2	38	3	2	6
Asarkina porcina			18	3	2	6
Betasyrphus serarius	1	1	41	3	2	6
Dasysyrphus albostriatus	6	2	16	2	3	6
Dasysyrphus bilineatus	5	2	43	3	2	6
Dasysyrphus tricinctus			12	2	3	6
Didea alneti	1	1	41	2	4	8
Didea fasciata	2	2	39	3	3	9
Dideoides coquiletti	1	1	66	2	2	4
Dideoides latus			42	2	2	4
Doros conopseus			4	2	3	6
Epistrophe grossulariae			2	2	4	8
Epistrophe nitidicollis			6	2	4	8
Episyrphus balteatus	59	6	max	3	3	9
Eriozona syrphoides			2	2	3	6
Ischyrosyrphus glaucius			6	2	3	6
Ischyrosyrphus laternarius			4	2	3	6
Leucozona lucorum			3	2	4	8
Melangyna compositarum			14	2	4	8
Melangyna lasiophthalma			7	2	3	6
Meliscaeva cinctella			15	3	4	12
Eupeodes confrater	1	1	2	2	2	4
Eupeodes corollae	32	4	28	3	3	9
Eupeodes luniger	1	1	3	3	4	12
Eupeodes nitens	24	5	52	2	3	6
Parasyrphus annulatus			14	2	3	6
Scaeva komabensis	1	1	10	2	2	4
Scaeva pyrastri	1	1	11	2	4	8
Sphaerophoria menthastri	649	6	max	2	3	6
Sphaerophoria rueppelli			3	2	3	6
Syrphus ribesii	3	2	24	2	4	8
Syrphus torvus	1	1	27	3	4	12
Syrphus vitripennis			53	3	4	12
Xanthogramma coreanum	2	2	19	2	1	2
Xanthogramma sapporense	1	1	14	2	2	4
Allobaccha apicalis			27	3	2	6
Baccha maculata	2	2	27	3	2	6
Chrysotoxum biguttatum			2	2	2	4

Chrysotoxum coreanum			4	2	1	2
Chrysotoxum festivum			10	3	3	9
Chrysotoxum grande			2	2	2	4
Chrysotoxum sapporense	2	2	19	2	2	4
Chrysotoxum shirakii			7	2	2	4
Melanostoma mellinum	37	6	62	2	4	8
Melanostoma scalare			23	3	3	9
Xanthandrus comtus	1	1	35	3	3	9
Platycheirus clypeatus			14	2	4	8
Platycheirus immaculatus	2	1	1	2	2	4
Platycheirus pennipes			1	2	2	4
Platycheirus scutatus			2	2	4	8
Platycheirus urakawensis	3	2	16	2	2	4
Paragus haemorrhous	206	6	56	3	3	9
Paragus quadrifasciatus	15	5	12	2	3	6
Pipiza austriaca			21	2	3	6
Pipiza flavimaculata	4	3	8	1	1	1
Pipiza inornata	2	2	0	1	1	1
Pipiza signata			14	3	3	9
Triglyphus primus	8	4	9	2	3	6
Cheilosia bombiformis			4	1	1	1
Cheilosia illustrata			8	2	3	6
Ferdinandea cuprea	2	2	19	2	3	6
Ferdinandea nigrifrons	2	1	5	1	3	3
Ferdinandea ruficornis	9	3	9	2	3	6
Rhingia laevigata	1	1	19	2	2	4
Callicera aenea			25	3	3	9
Volucella coreana			1	2	1	2
Volucella jeddona			10	2	2	4
Volucella linearis			32	1	1	1
Volucella matsumurai			9	1	1	1
Volucella nigricans			14	2	2	4
Volucella nigropicta			5	2	2	4
Volucella tabanoides			27	2	2	4
Volucella suzukii			6	1	1	1
Brachyopa testacea			1	2	3	6
Orthoneva karumaiensis			2	2	2	4
Sphegina clunipes			5	2	3	6
Pseudovolucella decipiens			10	2	2	4
Sericomyia dux			7	2	1	2
Eumerus iidai			1	2	4	8
Eumerus japonicus	12	5	13	2	2	4
Eumerus strigatus			1	2	4	8
Merodon kawamurai			2	1	1	1
Psilota brevicornis			1	1	1	1
Monoceromyia pleuralis			4	2	2	4
Primocerioides petri			4	2	2	4
Eristalinus sepulchralis			5	3	3	9
Eristalinus tarsalis			max	2	2	4
Eristalinus viridis			2	1	2	2
Eristalis arbustorum			max	3	4	12
Eristalis cerealis	1	1	max	3	2	6
Eristalis tenax	8	2	max	3	4	12
Phytomia zonata			40	3	2	6
Helophilus lunulatus			2	2	4	8
Helophilus trivittatus			1	2	3	6

Helophilus virgatus	12	5	max	2	2	4
Mallota analis			29	1	1	1
Mallota ambigua			5	1	1	1
Mallota dimorpha			16	2	2	4
Mallota tricolor			19	2	3	6
Mesembrius flavipes			7	2	2	4
Blera japonica			14	2	2	4
Macrozelima hervei			3	2	2	4
Matsumyia nigrofacies			11	2	2	4
Milesia undulata			3	2	2	4
Pterallastes unicolor			14	2	2	4
Spilomyia suzukii			18	2	2	4
Syritta pipiens	5	1	70	3	4	12
Temnostoma bombylans	1	1	17	3	3	9
Temnostoma vespiforme			6	2	4	8
Brachypalpus laphriformis			6	2	3	6
Chalcosyrphus sapporoensis	1	1	25	2	2	4
Xylota coquilletti	9	3	17	3	2	6
Xylota frontalis	40	8	29	1	1	1
Xylota ignava	1	1	0	2	3	6
Microdon auricomus	1	1	41	1	1	1
Microdon bifasciatus			3	1	1	1
Microdon caeruleus	7	3	4	2	2	4
Microdon japonicus			7	1	1	1
Microdon oitanus			8	1	1	1

Table S2. Flower flies (Syrphidae, Diptera) collected at the Gwangneung forest gap in South Korea. Habitat, G: gap, E: edge, F: forest, Sampling, M: Malaise trap, P: pitfall trap. Numbers in table represent the number of individuals collected. Species are arranged following Han and Choi (2001).

Species	Habitat			Sampling Method		Sampling Month						
	G	E	F	M	P	6	7	8	9	10	11	12
Allograpta javana		3		3				2	1			
Betasyrphus serarius	1				1						1	
Dasysyrphus albostriatus	6			1	5				1			5
Dasysyrphus bilineatus	3	2		5					1	4		
Didea alneti		1		1						1		
Didea fasciata	1	1		2						2		
Dideoides coquiletti		1		1			1					
Episyrphus balteatus	37	22		43	16	5	8	9	13	10	11	3
Eupeodes confrater		1		1					1			
Eupeodes corollae	31	1		4	28					3	18	11
Eupeodes luniger	1				1						1	
Eupeodes nitens	7	17		22	2				1	22	1	
Scaeva komabensis		1		1							1	
Scaeva pyrastri	1			1						1		
Sphaerophoria menthastri	589	60		631	18	29	84	50	164	310	12	
Syrphus ribesii	3			3					1	2		
Syrphus torvus	1			1						1		
Xanthogramma coreanum		2		2				1	1			
Xanthogramma sapporense		1		1						1		
Baccha maculata	2			2						2		
Chrysotoxum sapporense	2			2					2			
Melanostoma mellinum	30	7		37		1		2	16	18		
Xanthandrus comtus	1			1							1	
Platycheirus immaculatus		2		2		1	1					
Platycheirus urakawensis	2	1		3						3		
Paragus haemorrhous	130	76		206		9	41	46	80	28		2
Paragus quadrifasciatus	15	1		15				2	12	1		
Pipiza flavimaculata	3	1		4					4			
Pipiza inornata	2			2						2		
Triglyphus primus	7	1		8				1	5	2		
Ferdinandea cuprea	1		1	2			2					
Ferdinandea nigrifrons	2				2				2			
Ferdinandea ruficornis		9		9			9					
Rhingia laevigata	1				1				1			
Eumerus japonicus	7	5		12		3	3	3				3
Eristalis cerealis	1			1			1					

Eristalis tenax	7	1		1	7		1				2	5
Helophilus virgatus	2	9	1	9	3		3			9		
Syritta pipiens	5			4	1		4				1	
Temnostoma bombylans		1		1		1						
Chalcosyrphus sapporoensis	1			1		1						
Xylota coquilletti	8	1		9		2	1	4		2		
Xylota frontalis	7	28	5	40		2	36	2				
Xylota ignava		1		1		1						
Microdon auricomus	1			1								1
Microdon caeruleus	1	6		7			6	1				
Number of species	35	29	3	42	12	11	15	12	17	20	10	7
Number of individuals	919	263	7	1103	85	55	201	123	306	424	49	30

Reference

Han, H.-Y.; Choi, D.-S. *Diptera (Syrphidae): Economic Insects of Korea 15, Insecta Koreana Suppl. 22*; National Institute of Agricultural Science and Technology: Suwon, Korea, 2001.
